# Supplementary material for: When the hammer drops: Identification of knapping techniques in blade production based on a multi-scale study of knapping traces
Source: PLoS One. 2025 Aug 27;20(8):e0329848. doi: 10.1371/journal.pone.0329848 (PMC12385441; doi:10.1371/journal.pone.0329848)
Supplement: S1 Text — (DOCX) [file pone.0329848.s008.docx]

This document lists the microscopic traces considered in the visual analysis of the experimental blades and the attributes used to document them. The following general principles were also applied when recording the data:

- In cases where an attribute could not be determined, ‘ND’ was indicated.
- In cases where an attribute could not be recorded (e.g., because the knapping trace was absent), ‘NA’ was indicated.
- In cases where an attribute indicates the lateralization of a knapping trace, this lateralization was always determined with the dorsal face of the blade facing the observer, its platform facing upwards.

1. **Platform scarring**

- **Presence:** presence or absence of spontaneous scars. The values used are: ‘Yes’, and ‘No’.
- **N zones:** the relative number of zones where spontaneous scars are present. The values used are ‘Unique’ (one zone) and ‘Multiple’ (several zones).
- **Position:** the position of the spontaneous scars on both edges of the platform. The values used are ‘Anterior edge’, ‘Posterior edge’, and ‘Both edges’.
- **Lateralization:** the lateralization of spontaneous scars on the edges of the platform. The values used are ‘Left’, ‘Center’, and ‘Right’.
- **Organization:** the organization of the spontaneous scars. If only one scar is present, the value used is ‘Unique scar’. If several scars are present, the values used are ‘Isolated’ (the scars are far apart), ‘Close’ (the scars are close to each other but are not in contact), ‘Aligned’ (the scars are in contact), ‘Superimposed’ (there is an overlap of scars), and ‘Variable’ (several organizations are observed).
- **Morphology:** the morphology of the spontaneous scars. The values used are ‘Scalar’, ‘Quadrangular’, ‘Trapezoidal’, ‘Triangular’, ‘Crescent’, ‘Irregular’, and ‘Variable’.
- **Initiation:** the type of initiation of the spontaneous scars. The values used are ‘Cone’, ‘Bending’, ‘Mixed’ (for initiations between cone and bending), and ‘Variable’.
- **Initiation depth:** the depth of initiation of the spontaneous scars. The values used are ‘None’, ‘Limited’, ‘Pronounced’, and ‘Variable’.
- **State denticles:** the condition of the denticles on both sides of the spontaneous scar initiation zone. The values used are ‘Intact’, ‘Crushed’, ‘Blunted’, and ‘Variable’.
- **Termination:** the termination of the spontaneous scars. The values used are ‘Feather’, ‘Hinged’, ‘Step’, ‘Snap’, and ‘Variable’.

1. **Edge scarring**

- **Presence:** presence or absence of spontaneous scars. The values used are: ‘Yes’, and ‘No’.
- **N zones:** the relative number of zones where spontaneous scars are present. The values used are ‘Unique’ (one zone) and ‘Multiple’ (several zones).
- **Position:** the position of the spontaneous scars on both faces of the blade. The values used are ‘Dorsal face’, ‘Ventral face’, and ‘Both faces’.
- **Location:** the location of the spontaneous scars along the length of the blade. The values used are ‘Entire length’, ‘Proximal’, ‘Proximo-mesial’, ‘Mesial’, ‘Mesio-distal’, and ‘Distal’.
- **Lateralization:** the lateralization of spontaneous scars on the blade. The values used are ‘Left’, ‘Right’, and ‘Left & right’.
- **Organization:** the organization of the spontaneous scars. If only one scar is present, the value used is ‘Unique scar’. If several scars are present, the values used are ‘Isolated’ (the scars are far apart), ‘Close’ (the scars are close to each other but are not in contact), ‘Aligned’ (the scars are in contact), ‘Superimposed’ (there is an overlap of scars), and ‘Variable’ (several organizations are observed).
- **Morphology:** the morphology of the spontaneous scars. The values used are ‘Scalar’, ‘Quadrangular’, ‘Trapezoidal’, ‘Triangular’, ‘Crescent’, ‘Irregular’, and ‘Variable’.
- **Initiation:** the type of initiation of the spontaneous scars. The values used are ‘Cone’, ‘Bending’, ‘Mixed’ (for initiations between cone and bending), and ‘Variable’.
- **Initiation depth:** the depth of initiation of the spontaneous scars. The values used are ‘None’, ‘Limited’, ‘Pronounced’, and ‘Variable’.
- **State denticles:** the condition of the denticles on both sides of the spontaneous scar initiation zone. The values used are ‘Intact’, ‘Crushed’, ‘Blunted’, and ‘Variable’.
- **Termination:** the termination of the spontaneous scars. The values used are ‘Feather’, ‘Hinged’, ‘Step’, ‘Snap’, and ‘Variable’

1. **Cracks**

- **Presence:** the presence or absence of cracks. The values used are: ‘Yes’, and ‘No’.
- **N:** the relative number of cracks. The values used are ‘Unique’ and ‘Multiple’.
- **Distribution:** if there are several cracks, the relative distribution of cracks on the surface of the platform. The values used are ‘Concentrated’ (if all the cracks are in the same zone), and ‘Dispersed’ (if the cracks are not in the same zone).
- **Localization:** the relative localization of the cracks on the platform. The posterior edge of the platform was used as a reference. The values used are ‘Contact posterior edge’, ‘Close posterior edge’, ‘Distant posterior edge’, and ‘Variable’.
- **Dimensions:** the relative dimensions of the cracks. The values used are ‘Limited’, ‘Extended’, and ‘Variable’.
- **Associated crushing:** the presence or absence of a small crushed zone associated with the cracks. The values used are ‘Yes’, ‘No’, and ‘Variable’.
- **Types of cracks:** see Fig3.

1. **Cracks (anterior edge)**

- **Presence:** the presence or absence of cracks along the anterior edge of the platform. These cracks most likely formed during the preparation of the edge of the striking/pressure platform before the blade was detached. The values used are: ‘Yes’, and ‘No’.
- **N zones:** the relative number of zones along the anterior edge of the platform where cracks are present. The values used are ‘Unique’ and ‘Multiple’.

1. **Pre- and post-cleaning residues**

Pre-cleaning residues correspond to the knapping residues observed on the platform of a blade after its detachment. Once documented, these residues were removed by cleaning. The cleaning procedures used were adapted according to the nature of the residues and their resistance. In some cases, the residues (particularly organic residues) exhibited very pronounced adhesion, so certain platforms had to be cleaned in several phases. Cleaning was stopped when all the residues had been eliminated or when they had been reduced sufficiently to allow analysis of other types of traces (e.g. cracks, polishes and incisions) present on the platform that were not or only partially visible before cleaning. The cleaning of organic residues involved the use of a 3% and sometimes 10% hydrochloric acid (HCl) solution and an ultrasonic bath, for varying lengths of time depending on the resistance of the residues. When the mineral residues were too numerous, they were removed with an ultrasonic bath for 10 minutes. Copper residues were sufficiently limited that other traces could be observed, but we carried out several attempts to remove them using an ultrasonic bath and a 3% HCl solution, with limited results. Acetone was used for varying lengths of time in an attempt to remove the residue strip along the posterior edge of some blades. This strip was generally reduced but not completely removed. Post-cleaning residues correspond to what remained of the initial knapping residues after the cleaning procedure. We did not attempt to remove these remaining residues because they did not prevent the analysis of the other traces.

- **Presence:** the presence or absence of residues, before or after cleaning. The values used are: ‘Yes’, and ‘No’.
- **Distribution:** the relative distribution of residues on the surface of the platform. The values used are ‘Concentrated’ (if all residues are in the same zone), and ‘Dispersed’ (if the residues are not in the same zone).
- **Correlation with cracks:** the association of residues with cracks. The values used are ‘Associated’ (if residues are found inside the cracks), ‘Dissociated’ (if the residues are found outside the cracks), and ‘Partly associated’ (if the residues are found both inside and outside the cracks).
- **Morphology:** the general appearance of the residues. The values used are ‘Linear’, ‘Non-linear’, and ‘Variable’. This attribute was not recorded for post-cleaning residues, as the initial residues were modified after the cleaning process.
- **Incisions associated:** the presence or absence of incisions associated with the residues. The values used are ‘Yes’, and ‘No’.

1. **Pre-cleaning residue strip along posterior edge**

Only the presence or absence of a residue strip along the posterior edge of the platform was recorded. The values used are ‘Yes’, and ‘No’.

1. **Polishes**

- **Presence:** the presence or absence of polishes. The values used are ‘Yes’, and ‘No’.
- **N zones:** the relative number of zones where polishes are present. The values used are ‘Unique’ and ‘Multiple’.
- **Distribution:** in cases where several polish zones are present, the relative distribution of polishes on the surface of the platform. The values used are ‘Concentrated’ (if all polishes are in the same zone), and ‘Dispersed’ (if polishes are not in the same zone).
- **Density:** the relative interconnectedness of the polishes. The values used are ‘Low’, ‘Intermediate’, and ‘High’.
- **Localization:** the relative localization of the polishes on the platform. The posterior edge of the platform was used as a reference. The values used are ‘Contact posterior edge’, ‘Close posterior edge’, ‘Distant posterior edge’, and ‘Variable’.
- **Correlation with cracks:** the association between polishes and cracks. The values used are ‘Associated’ (if the polishes are inside the cracks), ‘Dissociated’ (if the polishes are outside the cracks), and ‘Partly associated’ (if the polishes are both inside and outside the cracks).
- **Morphology:** the general appearance of the polishes. The values used are ‘Linear’, ‘Non-linear’, and ‘Variable’.
- **Incisions associated:** the presence or absence of incisions associated with the polishes. The values used are ‘Yes’, and ‘No’.
- **Linear polishes - Orientation:** the orientation of the linear polishes in relation to each other. The values used are ‘Parallel’, and ‘Secant’.
- **Linear polishes - Regularity:** the regularity of the linear polishes. The values used are ‘Continuous’ (the “lines” formed by the linear polishes have no interruptions), ‘Discontinuous’ (the “lines” formed by the linear polish have one or more clear interruptions), and ‘Variable’ (both situations are observed).
- **Linear polishes - Morphology:** the appearance of the linear polishes. The values used are ‘Rectilinear’, ‘Curved’, and ‘Variable’.
- **Linear polishes - Lateralization curve:** if the linear polishes are curved, the lateralization of the curve. The values used are ‘Left’ (if the direction of the curve is towards the left edge of the blade), and ‘Right’ (if the direction of the curve is towards the right edge of the blade).

1. **Polishes (anterior edge)**

- **Presence:** the presence or absence of polishes along the anterior edge of the platform. These polishes most likely formed during the preparation of the edge of the striking/pressure platform before the blade was detached. The values used are: ‘Yes’, and ‘No’.
- **N zones:** the relative number of zones along the anterior edge of the platform where polishes are present. The values used are ‘Unique’ and ‘Multiple’.

1. **Pre-cleaning residues on the dorsal and ventral faces**

Unlike the residues present on the platform of a blade, the residues present on its dorsal or ventral faces were not cleaned and, therefore, we only observed and documented them before cleaning.

- **Presence:** the presence or absence of residues on the dorsal and ventral faces of the blade. The values used are ‘Yes’, and ‘No’.
- **Material category:** the general nature of the residues located on the dorsal and ventral faces of the blade. The values used are: ‘Mineral’, ‘Organic’, ‘Copper’, ‘Mineral + organic’, ‘Copper + organic’, and ‘Mineral + copper’.
- **Location:** the location of the residues along the length of the blade. The values used are ‘Entire length’, ‘Proximal’, ‘Proximo-mesial’, ‘Mesial’, ‘Mesio-distal’, and ‘Distal’. This attribute was not recorded for residues located on the dorsal face of the blade, as we were primarily interested in determining where the friction between the blade and the core had occurred, which had led to the deposition of residues on the ventral face.
- **Morphology:** the general appearance of the residues. The values used are ‘Linear’, ‘Non-linear’, and ‘Variable’. This attribute was not recorded for residues located on the dorsal face of the blade, as we were primarily interested in determining whether friction between the blade and the core resulted in the deposition of linear or non-linear residues on the ventral face.
