# Supplementary material for: When the hammer drops: Identification of knapping techniques in blade production based on a multi-scale study of knapping traces
Source: PLoS One. 2025 Aug 27;20(8):e0329848. doi: 10.1371/journal.pone.0329848 (PMC12385441; doi:10.1371/journal.pone.0329848)
Supplement: S1 Fig — Review of the knapping features used in the literature and their diagnostic value. (DOCX) [file pone.0329848.s009.docx]

**S1a – Number of studies examined per decade**

**S1b – Number of studies examined per MFA and CT used**

**S1c – Distribution of the number of attributes according to the number of studies in which they were used**

**S1d – Distribution of the number of attributes according to the number of times they were used**
