# Supplementary figures and images for: When the hammer drops: Identification of knapping techniques in blade production based on a multi-scale study of knapping traces
Source: PLoS One. 2025 Aug 27;20(8):e0329848. doi: 10.1371/journal.pone.0329848 (PMC12385441; doi:10.1371/journal.pone.0329848)

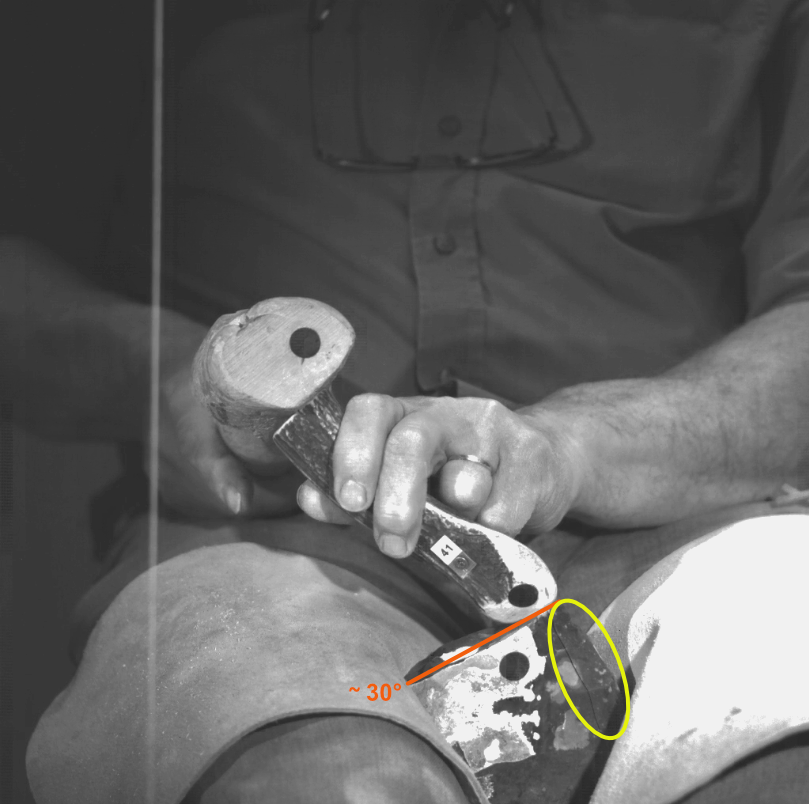

Supplement: S2 Fig — Note that the knapper has positioned the core so that the striking platform is inclined at around 30° to the ground. The way he holds the core allows the proximo-mesial part of the blade (yellow circle) to be free of any constraint. This part can therefore tilt forward after the blade has been extracted, but as its distal part is still held firmly between the core and the knapper’s thigh, the blade returns towards the core and hits it. This specific position creates spontaneous scars with intact denticles on the proximo-mesial part of the blade, and spontaneous scars with crushed denticles on its distal part. (TIFF) [file pone.0329848.s010.tiff]

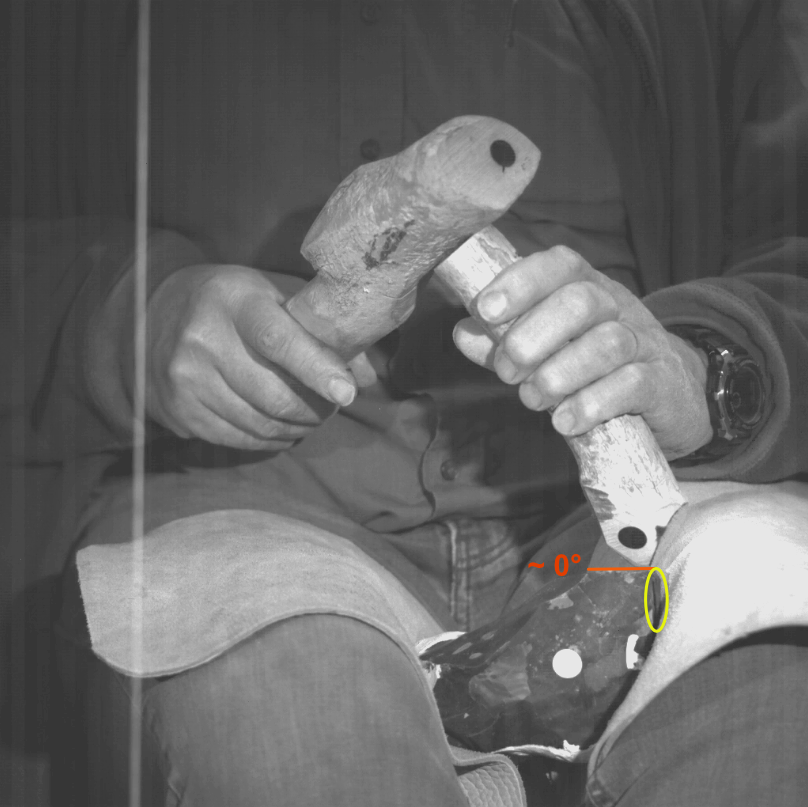

Supplement: S3 Fig — Note that the knapper has positioned the core so that the striking platform is almost parallel to the ground. In this case, the entire length of the flaking surface is firmly maintained against the knapper’s thigh and only the proximal part of the blade (yellow circle) can barely move away from the core. In this position, there is no significant friction between the blade and the core, so that spontaneous scars show only intact denticles. (TIFF) [file pone.0329848.s011.tiff]

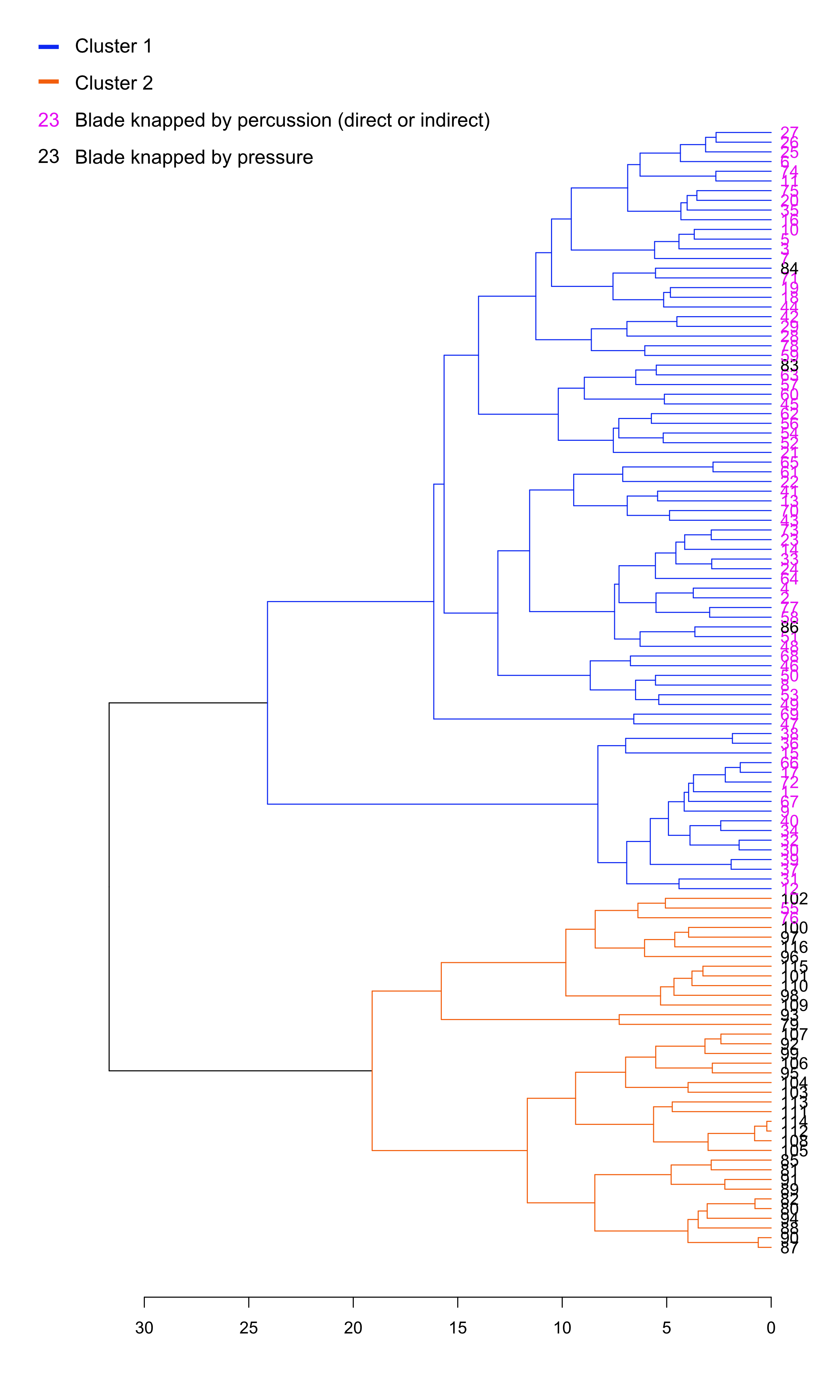

Supplement: S4 Fig — (TIFF) [file pone.0329848.s012.tiff]

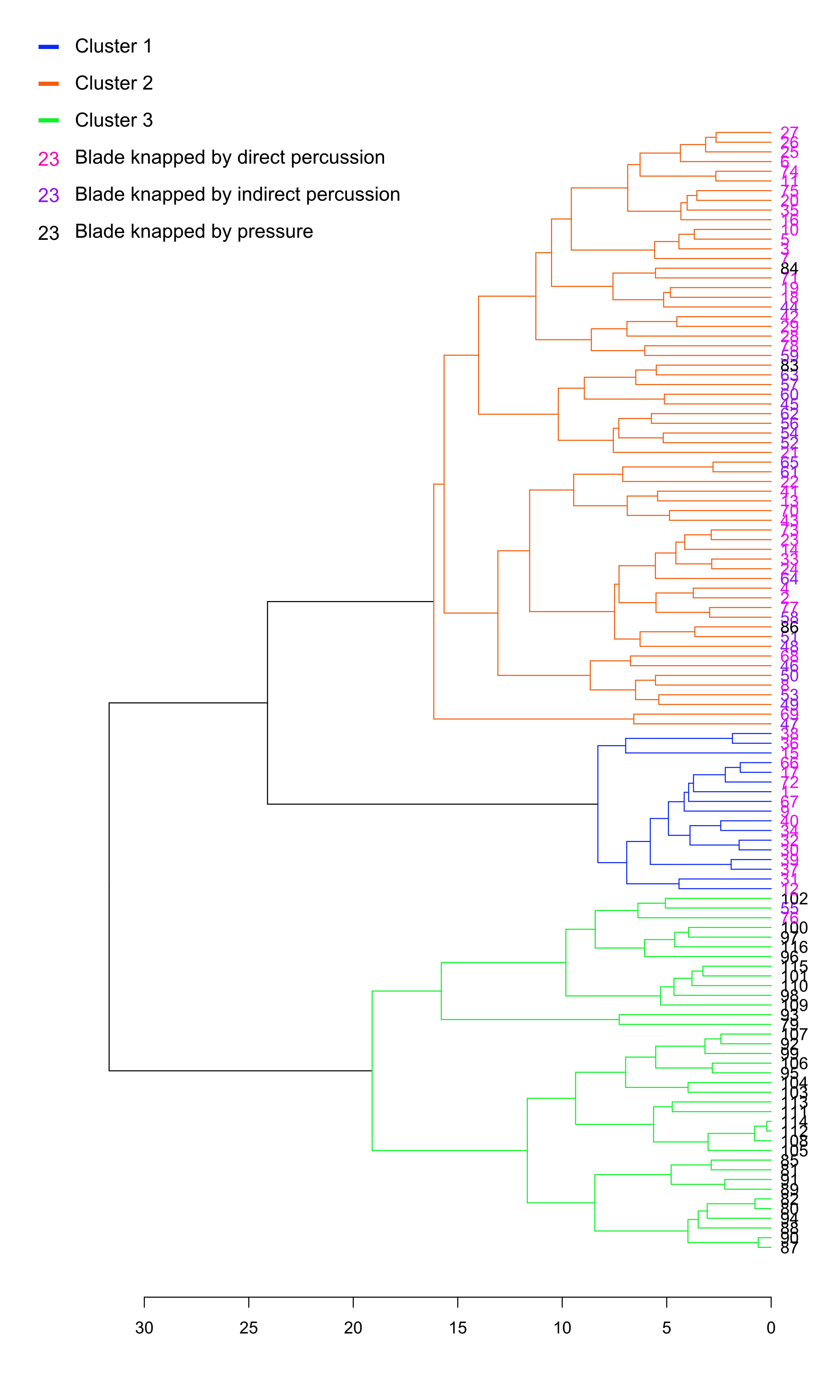

Supplement: S5 Fig — (TIFF) [file pone.0329848.s013.tiff]

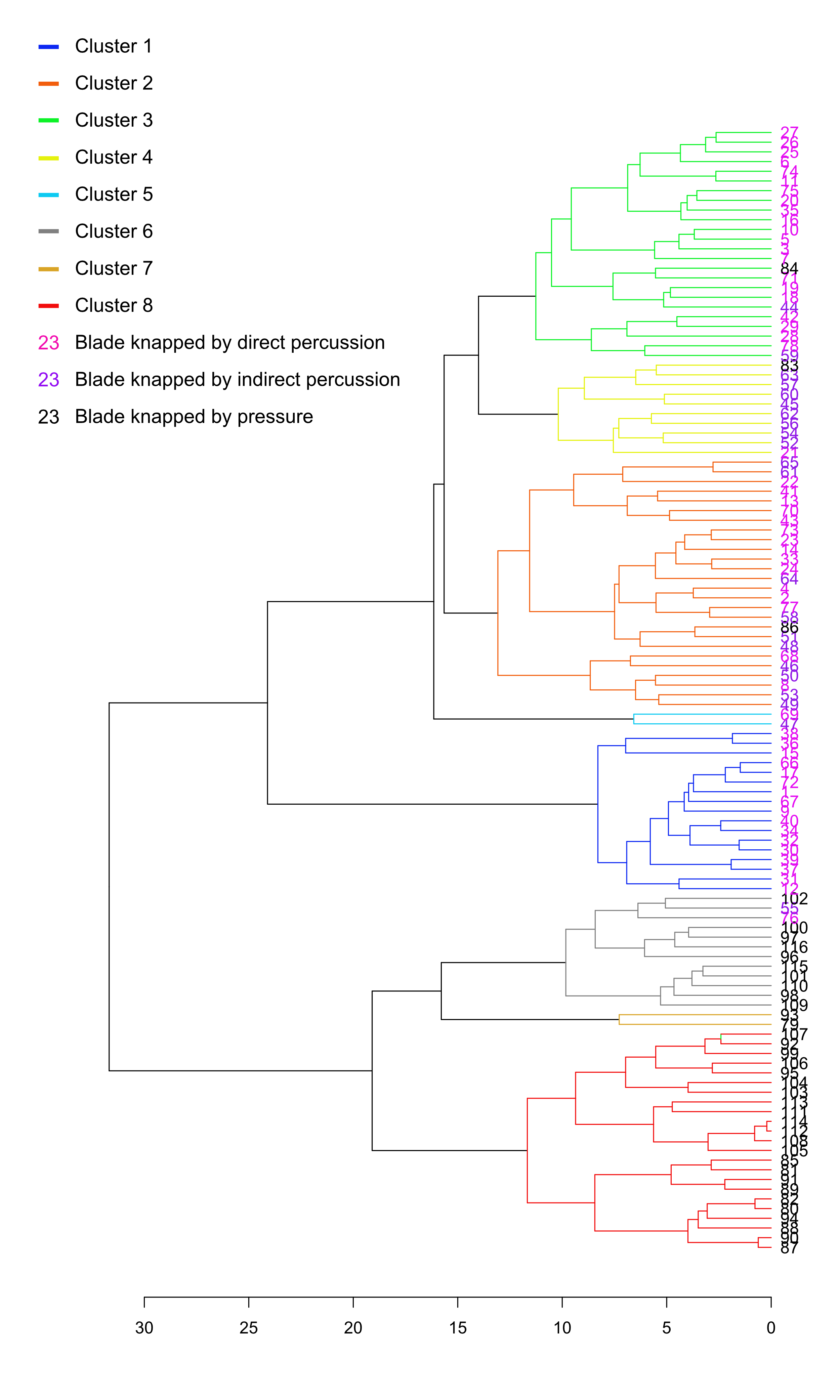

Supplement: S6 Fig — (TIFF) [file pone.0329848.s014.tiff]

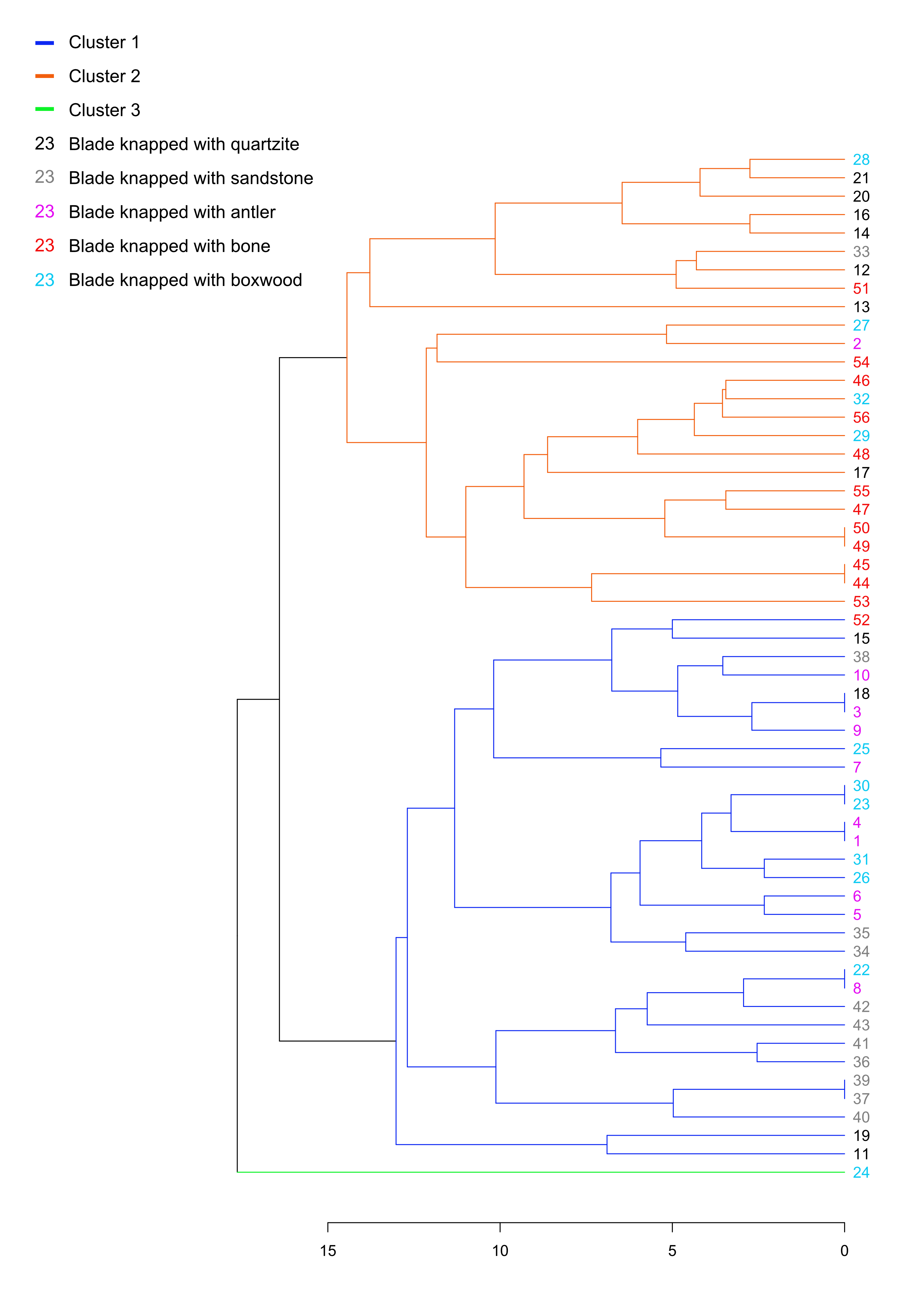

Supplement: S7 Fig — (TIFF) [file pone.0329848.s015.tiff]

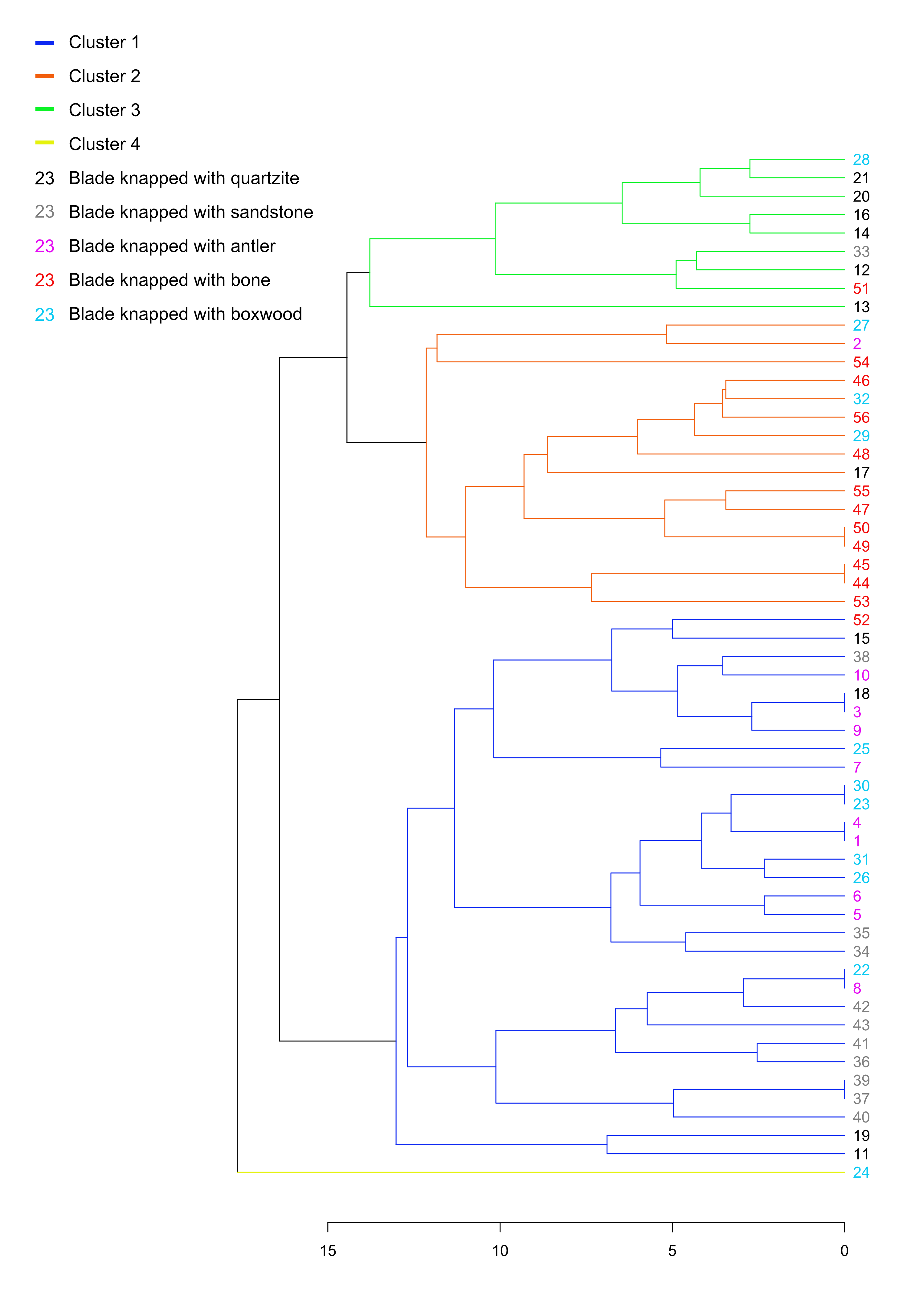

Supplement: S8 Fig — (TIFF) [file pone.0329848.s016.tiff]

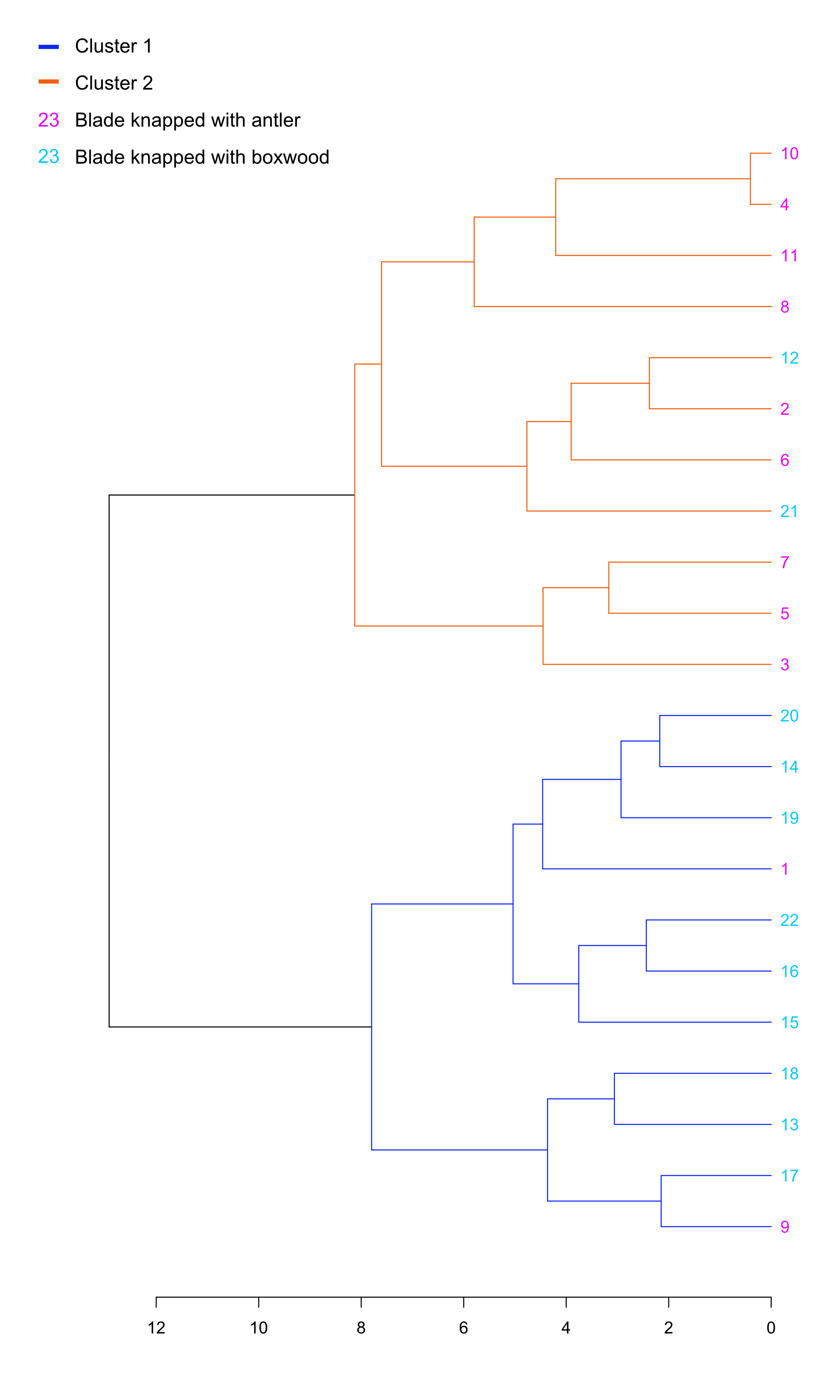

Supplement: S9 Fig — (TIFF) [file pone.0329848.s017.tiff]

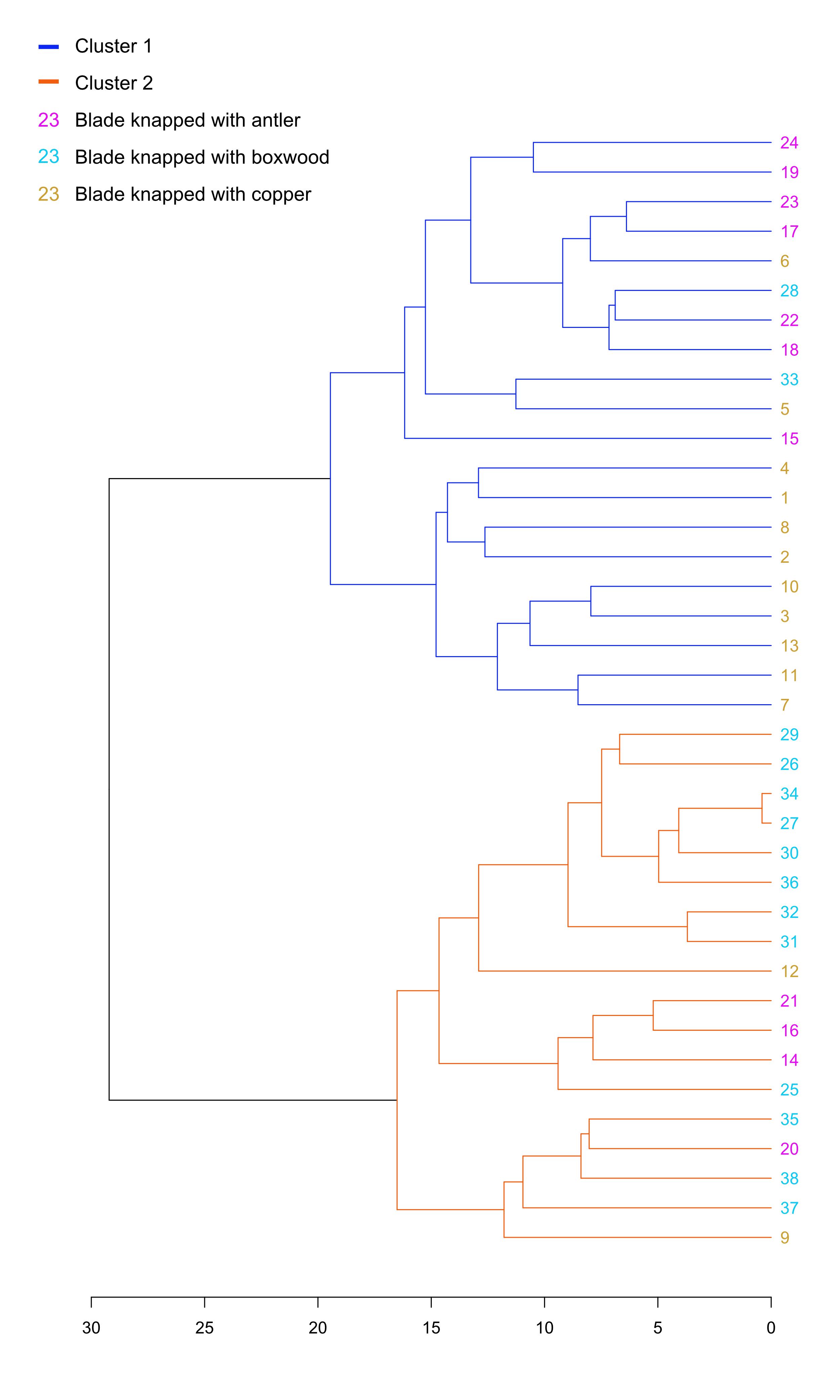

Supplement: S10 Fig — (TIFF) [file pone.0329848.s018.tiff]

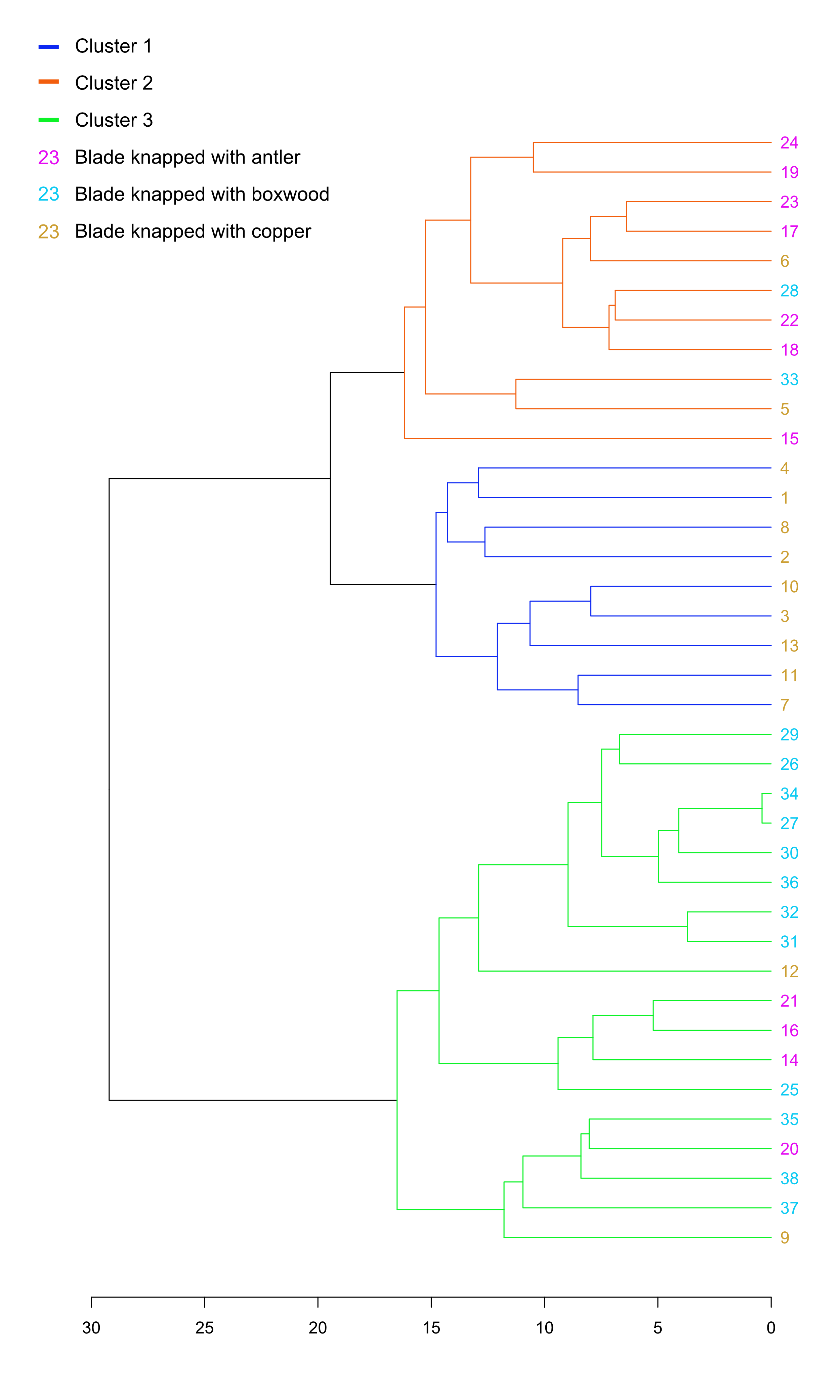

Supplement: S11 Fig — (TIFF) [file pone.0329848.s019.tiff]

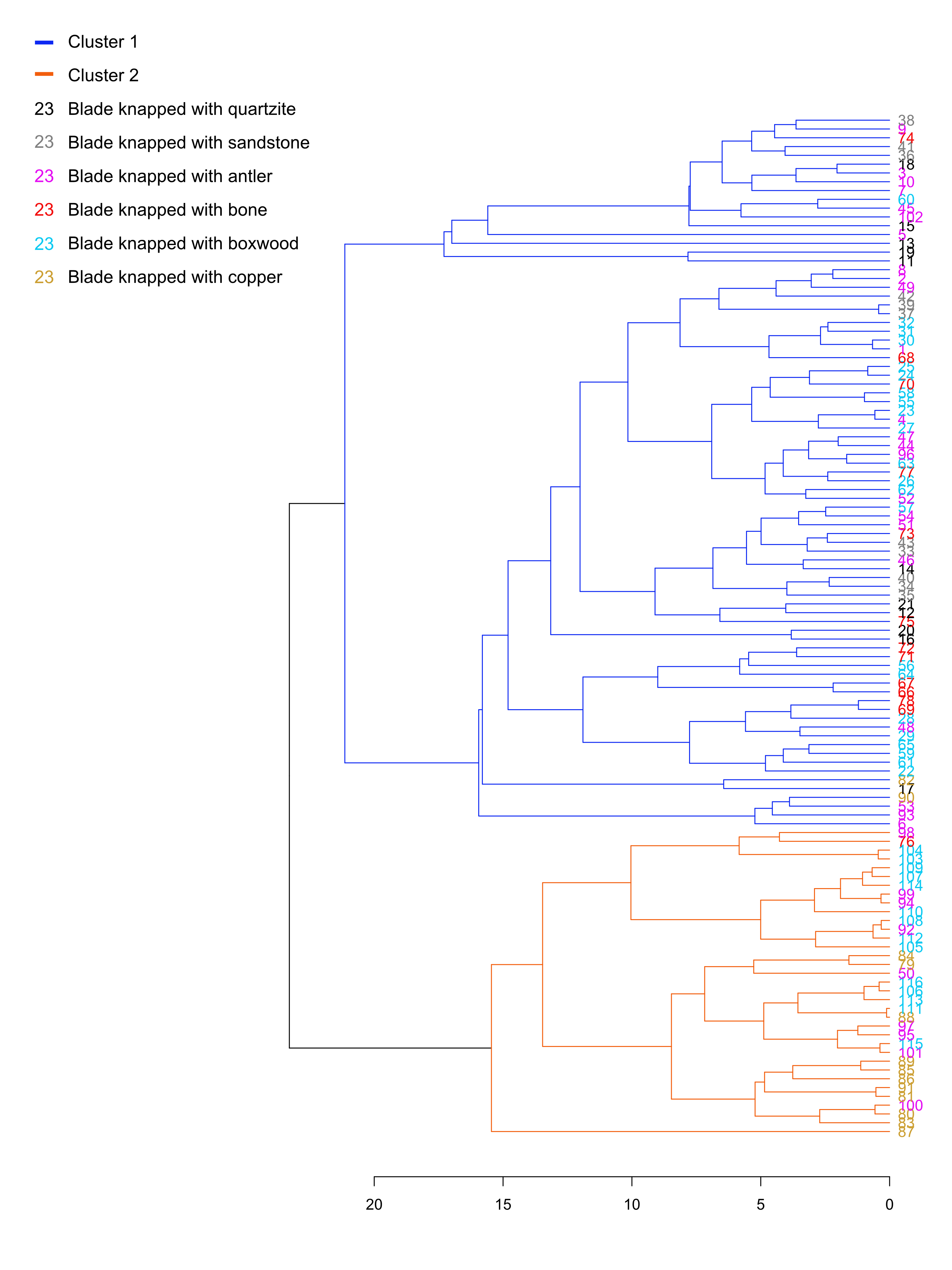

Supplement: S12 Fig — (TIFF) [file pone.0329848.s020.tiff]

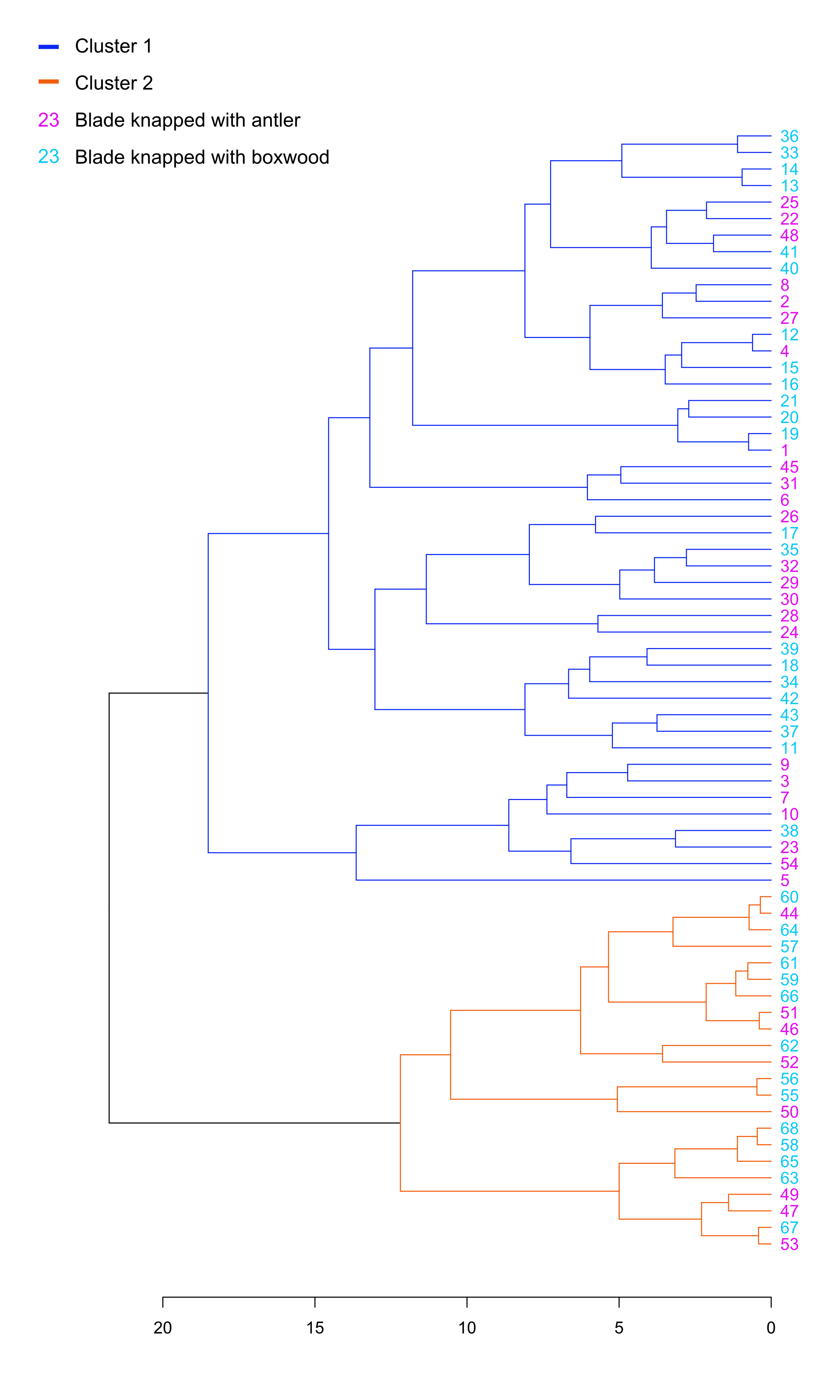

Supplement: S13 Fig — (TIFF) [file pone.0329848.s021.tiff]
